# Overexpression and altered glycosylation of MUC1 in malignant mesothelioma

**DOI:** 10.1038/sj.bjc.6604340

**Published:** 2008-04-29

**Authors:** J Creaney, A Segal, G Sterrett, M A Platten, E Baker, A R Murch, A K Nowak, B W S Robinson, M J Millward

**Affiliations:** 1 National Research Centre for Asbestos Related Diseases, Western Australian Institute of Medical Research, University of Western Australia, Perth, Western Australia, Australia; 2 Australian Mesothelioma Tissue Bank, Sir Charles Gairdner Hospital, Perth, Western Australia, Australia; 3 Department of Anatomical Pathology, PathWest, Queen Elizabeth II Medical Centre, Perth, Western Australia, Australia; 4 Department of Pathology, University of Western Australia, Perth, Western Australia, Australia; 5 PathWest, Queen Elizabeth II Medical Centre, Perth, Western Australia, Australia; 6 PathWest Department of Cytogenetics, PathWest, King Edward Memorial Hospital, Perth, Western Australia, Australia; 7 Department of Pediatrics, University of Adelaide, Adelaide, Australia; 8 School of Surgery and Pathology, University of Western Australia, Perth, Western Australia, Australia; 9 School of Medicine and Pharmacology, University of Western Australia, Perth, Western Australia, Australia; 10 Department of Medical Oncology, Sir Charles Gairdner Hospital, Perth, Western Australia, Australia; 11 Department of Respiratory Medicine, Sir Charles Gairdner Hospital, Perth, Western Australia, Australia

**Keywords:** mesothelioma, MUC-1, EMA, diagnosis

## Abstract

Current interest in the MUC1/EMA mucin relates to its role in malignancy, and its potential as a therapeutic target. MUC1/EMA expression has been observed in the majority of epithelioid mesotheliomas. However, little is known of the characteristics of MUC1/EMA in mesothelioma. Herein, we studied the cell surface and soluble expression of the MUC1/EMA glycoprotein, and determined the mRNA and genomic expression profiles in mesothelioma. We found that the anti-MUC1 antibody, E29, was the most diagnostically useful of seven antibody clones examined with a sensitivity of 84% (16 out of 19 cases) and no false positive results. MUC1 mRNA expression was significantly higher in mesothelioma samples than in benign mesothelial cells. No amplification of the MUC1 gene was observed by FISH. Seven of 9 mesothelioma samples expressed MUC1-secreted mRNA isoform in addition to the archetypal MUC1/transmembrane form. CA15.3 (soluble MUC1) levels were significantly higher in the serum of mesothelioma patients than in healthy controls but were not significantly different to levels in patients with benign asbestos-related disease. CA15-3 in effusions could differentiate malignant from benign effusions but were not specific for mesothelioma. Thus, as in other cancers, alterations in MUC1 biology occur in mesothelioma and these results suggest that specific MUC1 characteristics may be useful for mesothelioma diagnosis and should also be investigated as a potential therapeutic target.

The human MUC1 gene encodes a protein which undergoes glycosylation and has been variously referred to as epithelial membrane antigen (EMA), human milk fat globule antigen, breast-cancer associated DF3 antigen, polymorphic epithelial mucin, sialomucin, CD227, episialin and CA15-3 (Taylor-Papadimitriou *et al*, 2002). The major isoform of the MUC1 gene consists of a high molecular weight glycosylated extracellular domain, a variously amplified 20 amino acid repeat sequence (designated the variable number tandem repeat (VNTR) region), and a hydrophobic 31 amino acid transmembrane domain with a 69 amino acid cytoplasmic tail (Gendler and Spicer, 1995). This isoform of MUC1, generally designated simply as MUC1, or MUC1/transmembrane (MUC1-TM), is expressed on the apical cell surface of the normal glandular epithelium of many tissues, and also on various haematopoietic cells. The MUC1 gene products have been intensely investigated and alterations in expression, splicing patterns, secretion and glycosylation patterns have been observed in many malignant conditions. These malignancy-associated variations suggest that MUC1 may be an attractive target for anticancer therapies.

Recent studies of MUC1-targeted therapies in lung, prostate and ovarian malignancies (Loveland *et al*, 2006; North *et al*, 2006) show therapeutic promise. As the majority of epithelioid mesotheliomas demonstrate strong MUC1/epithelial membrane antigen (EMA) positivity by immunohistochemistry (Ordonez, 2003) similar anti-MUC1 therapeutic strategies may have potential in mesothelioma. Malignant mesothelioma is an aggressive tumour with a median survival of less than 12 months and limited treatment options (Robinson *et al*, 2005).

Although controversial, EMA staining has a role in the diagnosis of mesothelioma, particularly by effusion cytology distinguishing malignant mesothelioma cells from benign reactive mesothelial cells (Wolanski *et al*, 1998; Whitaker, 2000). The controversy is in part due to the anti-EMA antibody clone used in studies (Saad *et al*, 2005) as a variety of antibodies have been generated against the various heterogeneous MUC1 protein and glycoprotein isoforms that have been found in different tissues and malignant states.

In this paper, we have examined various aspects of the MUC1 molecule in malignant mesothelioma effusion and tissue samples to investigate its potential diagnostic role, mRNA expression, splice variants, and used fluorescence *in situ* hybridisation (FISH) to detect gene amplification. In addition, we have examined levels of CA15-3 (a secreted form of MUC1) in serum and effusion supernatant. These studies aim to provide a baseline analysis of mesothelioma-associated MUC1 isoforms, and also to determine which mesothelioma-specific features of MUC1 may be of potential diagnostic and therapeutic relevance in this disease.

## MATERIALS AND METHODS

### Patients, samples and controls

Serum, pleural effusion and surgically-excised tumour tissue samples were collected from patients following written informed consent. All biospecimens were provided by the Australian Mesothelioma Tissue Bank, a member of the ABN-oncology group, which is supported by the National Health and Medical Research Council, Australia. This study was approved by the human research ethics committees of Sir Charles Gairdner and Hollywood Hospitals, Perth, Western Australia. The final diagnosis in all patients was confirmed by pathologists experienced in the diagnosis of mesothelioma and included clinical follow-up of all cases until death or to last citation in the Public Hospital database system (iSoft Clinical Manager) to confirm that the clinical pattern matched the diagnosis. Mesotheliomas were classified as epithelial, sarcomatoid, mixed or, in cases where diagnosis was made on the basis of immunocytology and there was no histological follow-up, as unspecified.

Normal mesothelial cells were recovered from pericardial fluid obtained from patients undergoing intrathoracic surgery as described previously (Holloway *et al*, 2006). Serum samples were collected from healthy volunteers, and from patients with the asbestos-related lung disease, asbestosis, and the asbestos-related pleural disease, pleural plaques. Pleural effusion samples were collected from patients with effusions caused by nonmesothelioma malignancy. Effusions were classified as benign (i.e., nonmalignant) on the basis of cytological and immunohistochemical features, and further classified as exudates or transudates on the basis of Light's criteria (Light *et al*, 1972). Effusions were classified as being associated with an infection if micro-organisms were detected in the fluid. Patient characteristics are listed in Table 1.

### Immunohistochemistry

A tissue microarray was constructed by the Western Australian Research Tissue Network (Perth, Western Australia) from archival paraffin blocks. The tissue microarray contained effusion cell pellets from 20 cases of malignant mesothelioma and 16 cases of benign-reactive pleural effusions. Immunohistochemistry was performed using standard techniques. Briefly, sections were deparaffinised with xylene and rehydrated in a graded series of ethanol. Antigen-retrieval was performed for 10 min at high temperature in citrate buffer. Sections were incubated with anti-EMA clones for 60 min and washed in PBS. Immunodetection was performed using Envision+ Dual link detection system (Dako, Glostrup, Denmark). For negative controls, the primary antibody was omitted. Antibodies were purchased from various suppliers (Table 2). Some of these antibodies had been previously characterised by workshops organised by the International Society of Oncodevelopmental Biology and Medicine (ISOBM) in terms of the epitope recognised within the VNTR and whether that recognition was dependent upon the glycosylation status of the epitope. These antibodies were assigned to various classes or clusters by the ISOBM workshops based upon their staining profile on normal breast tissue samples (summarised in Table 2) (Cao *et al*, 1998; Hanisch, 1998).

Staining was assessed by three observers independently (JC, AS and GS). A positive result was defined as the presence of membranous staining on tumour cells. Staining intensity was graded semi-quantitatively as negative, equivocal (+/−), weak (1+), moderate (2+) or strong (3+). Moderate and strong positivity was only assigned where the majority of cells showed positive staining. Sensitivity was calculated as the total number of moderately and strongly stained mesothelioma samples divided by the total number of mesothelioma samples. Specificity was calculated as the number of negative benign control samples divided by the total number of benign control samples. False positive rate was calculated as the number of moderately and strongly stained benign samples divided by the total number of benign samples.

### Quantitative PCR

RNA was extracted using Rneasy kits (Qiagen, Clifton Hill, Victoria, Australia), following the manufacturer's protocol. cDNA was generated in a standard reverse transcriptase reaction using oligo dT to prime Superscript II (Invitrogen, Mt Waverly, Victoria, Australia). Quantitative PCR was performed with specific primer sets (MUC1 forward 5′-AGACGTCAGCGTGAGTGATG-3′; reverse 5′-GACAGCCAAGGCAATGAGAT-3′) (Ohuchida *et al*, 2006) on the iCycler iQ Real Time Detection System (BioRad, Gladesville, New South Wales, Australia) using the QuantiTech SYBR Green PCR Kit (Qiagen). The primers correspond to nucleotides 4757–5017 of the human polymorphic epithelial mucin gene (GeneBank Accession number M61170) and amplify the MUC1-TM product. The relative expression of MUC1-TM was calculated using the standard *delta C*_t_ formula as follows: 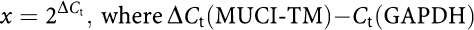


### Fish

4 *μ*m sections from the tissue microarray were deparaffinised, dehydrated, microwave treated in citrate buffer (pH 6.0) for 10 min, digested in pepsin solution (4 mg ml^−1^ in 0.01N HCl) for 7 min at 37°C, rinsed in 0.3 M sodium chloride, 30 mM sodium citrate (pH 7.0) at room temperature for 5 min. Dual-probe hybridisation was performed using three SpectrumGreen-labeled BAC clones which encompass the MUC1 gene at 1q22, plus a SpectrumOrange-labeled chromosome 1 centromeric probe (Vysis, Downers Grove, IL, USA) as a control. Seventeen mesothelioma samples were available for FISH analysis.

### Conventional PCR

Conventional PCR was performed with specific primer sets designed for different splice forms as described by (Obermair *et al*, 2002) and using Taq DNA polymerase (Qiagen). The primer pair Muc1-F (GCACTCACCATAGCACG) and Muc1-R (GGCCAGAGTCAATTGTAC) distinguish between MUC1-TM and a secreted MUC1 isoform, containing the VNTR but lacking the transmembrane domain and cytoplasmic tail, MUC1/secreted (SEC). PCR products were separated by electrophoresis on 3.5% agarose gel and visualised under UV light with ethidium bromide.

### Measurement of CA15-3

CA15-3 levels were determined using the IMMULITE 2000 BR-MA (CA15-3) assay (Diagnostic Products Corporation, Los Angeles, CA, USA) according to the manufacturer's instructions. The assay is a sandwich ELISA utilising the monoclonal antibodies 115D8 and DF3. A value above 53 kU l^−1^ was considered to be outside the normal range of healthy persons. All assays were performed on coded samples by technical staff unaware of the patient's diagnosis.

### Statistics

To test for statistically significant differences biomarkers were transformed to the logarithmic scale on which normal theory statistical estimates (mean, s.d.) and tests (*t*-tests) were applied.

## RESULTS

### EMA staining by Immunohistochemistry

**Figure 1:**
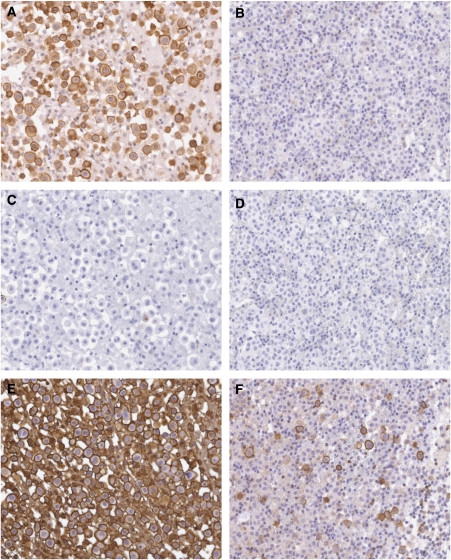
Immunohistochemical staining for MUC1/EMA on sections of formalin-fixed paraffin-embedded cell pellets from pleural fluid specimens. (**A**, **C** and **E**) are cells from a patient with mesothelioma. (**B**, **D** and **F**) are benign reactive mesothelial cells from a patient with a nonmalignant effusion. (**A** and **B**) were stained with the anti-EMA clone E29; (**C** and **D**) with the VU2G7 clone and (**E** and **F**) with the Mc5 clone.

The pattern of staining for all antibodies examined was similar, with accentuated staining detectable on the cell membrane (Figure 1). The sensitivity of the different antibody clones for mesothelioma effusion samples ranged from 25% for the VU2G7 and VU4H5 clones to 100% for the Mc5 clone. The E29 clone reacted with 16 (of 19) specimens (Table 3). Most of the antibodies studied were highly specific for malignant cells with no moderate or strongly stained cells being observed in up to 16 specimens of benign reactive effusion studied (Table 3). However the Mc5 clone reacted strongly with over half of the nonmalignant samples (57% false-positive rate). The MA695 clone had weak or equivocal staining on two thirds of these control samples (Table 3). The pattern of reactivity of the Mc5 clone was membrane accentuated in both the malignant and benign specimens.[Table tbl4]

### MUC1 mRNA is overexpressed in mesothelioma cells

**Figure 2:**
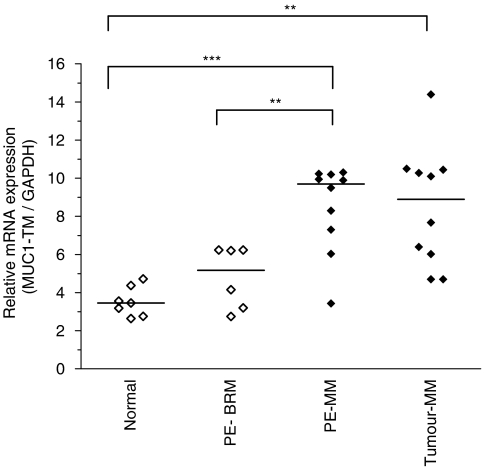
Relative expression of full length, transmembrane MUC1-TM normalised to GAPDH determined by quantitative PCR. Mean results for individual samples are indicated by diamonds, open diamonds are nonmalignant samples and closed diamonds are malignant samples. Mean levels for each group are indicated by the horizontal bar. Expression was determined in normal mesothelial cells derived from pericardial cells; cells from benign reactive pleural effusions (PE-BRM); mesothelioma samples from pleural effusions (PE-MM); and 10 mesothelioma tumour samples (Tumour-MM). ^***^P<0.0001; ^**^P<0.005.

The relative expression of the archetypal, full-length, transmembrane MUC1 isoform, MUC1-TM, normalised to GAPDH, was significantly greater in the cells from pleural effusion (*P*<0.0001) and surgically-excised tumour (*P*=0.001) mesothelioma samples than that in normal mesothelial cells (Figure 2). Median expression levels were two to threefold greater in malignant mesothelioma samples than in normal mesothelial cells. Expression of MUC1-TM in cells from mesothelioma effusions were significantly greater (*P*<0.005) than in effusions with benign reactive mesothelial cells. This overexpression was not due to gene amplification as there was no amplification of MUC1 by FISH (data not shown). There was no significant difference in relative levels of MUC1-TM between normal mesothelial cells and from cells obtained from patients with effusions of nonmalignant origin (Figure 2).

### Alternative splice forms of MUC1

**Figure 3:**
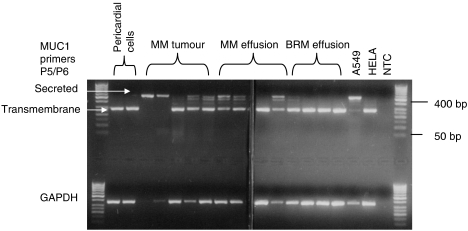
PCR products amplified from MUC1 transmembrane and MUC1-secreted isoforms from nonmalignant pericardial cells (*n*=2), malignant mesothelioma (MM) tumours (*n*=5), MM pleural effusion samples (*n*=4), nonmalignant effusions containing benign reactive mesothelial cells (BRM) (*n*=4), and as controls the lung adenocarcinoma cell line (A549), the cervical carcinoma cell line (HELA) and a no template control (NTC). Outside lanes contain 50 base pair ladder. Bottom panel shows glyceraldehyde-3-phosphate dehydrogenase PCR products from duplicate samples, processed in parallel.

Conventional PCR using primers distinguishing MUC1-TM from a secreted isoform of MUC1 which lacks the transmembrane region, MUC1-SEC, demonstrated that approximately half of the samples from malignant mesothelioma cells either from solid tumour or from effusions expressed MUC1-SEC in addition to the transmembrane form (Figure 3). Normal mesothelial cells, either from pericardial fluid or from benign reactive effusions predominately expressed the full length MUC1-TM isoform.

### Serum CA15-3 level

**Figure 4:**
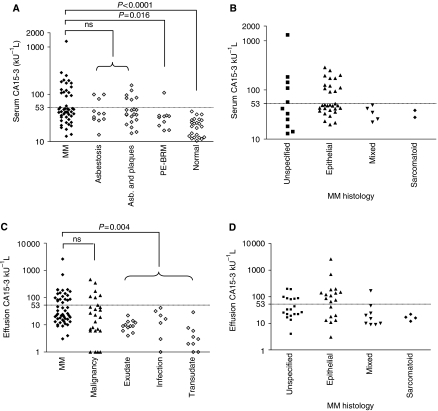
CA15-3 in mesothelioma patients and controls (**A**) Serum CA15-3 levels were determined in duplicate and individual patient values are plotted on the graph. Serum CA15-3 were plotted for malignant mesothelioma (MM) patients, individuals who had asbestosis and who had asbestosis and pleural plaques (asb. and plaques), patients with effusions containing benign reactive mesothelial cells (PE-BRM) and for control, healthy individuals with no documented asbestos exposure. (**B**) CA15-3 levels in mesothelioma patients segregated by tumour histology. Mesothelioma patients were characterised by the histology of the tumour (sarcomatoid, mixed histology or epithelial), or in those cases where diagnosis was made without histology being reported (unspecified). (**C**) CA15-3 levels were determined in effusions from mesothelioma patients (closed diamonds), from individuals with malignant effusions not due to mesothelioma (closed triangles), and from nonmalignant effusions of an exudative, or transudate nature or relating to an infection (open diamonds). (**D**) Pleural effusion CA15-3 of patients with malignant mesothelioma further characterised by the histology of the tumour (sarcomatoid, biphasic, epithelial or unspecified). The manufacturer defined upper limit of normal concentration (53 kU l^−1^) is depicted by the hashed horizontal line.

The median concentration of CA15-3 in the serum of patients with malignant mesothelioma was 57.4±2.5 kU l^−1^ (range, 13–1321 kU l^−1^); 35% (17 out of 49) of mesothelioma patients had serum CA15-3 above the upper limit of normal (53 kU l^−1^) (Figure 4A). Though patient numbers were limited, mesothelioma patients with a sarcomatoid or mixed histology had lower serum concentrations of CA15-3 than mesothelioma patients with a predominately epitheliod histology (Figure 4B).

Median CA15-3 concentration in patients with benign asbestos-related disease (either asbestosis and/or pleural plaques) was 38±6 kU l^−1^ (range, 14–155 kU l^−1^); 28% (9 out of 32) of patients with asbestos-related benign disease had CA15-3 levels above the upper limit of normal. There was no significant difference in serum CA15-3 between patients with mesothelioma and patients with benign asbestos-related lung and pleural disease. One of 10 patients with non-malignant pleural effusions examined had elevated serum CA15-3. Median CA15-3 concentration in the serum of healthy controls was 22±2 kU l^−1^ (range, 9–43 kU l^−1^). Median concentrations of CA15-3 were significantly higher in the serum of mesothelioma patients compared to patients with benign effusions (*P*=0.016) and normal controls (*P*<0.0001) (Figure 4A).

### Effusion CA15-3 level

Levels of CA15-3 in pleural effusions of patients with malignant mesothelioma ranged from undetectable to 2614 kU l^−1^, with a median of 36.9±3.9 kU l^−1^. Approximately 38% (20 out of 52) of mesothelioma patients had CA15-3 levels above the upper limit of normal (53 kU l^−1^) (Figure 4C). Mesothelioma patients with sarcomatoid histology had lower levels of CA15-3 in their effusion than mesothelioma patients with predominately epitheliod histology, although there was no significant difference between the groups (Figure 4D).

The median CA15-3 concentration in the effusions of patients with nonmesothelioma malignancies was 24±6 kU l^−1^ (range, 13–191 kU l^−1^); CA15-3 was elevated in 24% (6 out of 25) of effusions from these patients. There was no significant difference in the effusion CA15-3 levels between the groups of patients with mesothelioma or other malignancy. Median CA15-3 in effusions of nonmalignant origin was 8.5±2.9 kU l^−1^ (range from undetectable to 42 kU l^−1^). CA15-3 levels were statistically significantly higher in the effusions of patients with mesothelioma than those with benign effusions (*P*=0.004) (Figure 4C).

## DISCUSSION

Normal/reactive nonmalignant mesothelial cells express predominantly the full-length tandem-repeat containing MUC1-TM, which can be detected on the cell membrane by the Mc5 antibody. In cases of malignant mesothelioma there is an increase in the total quantity of MUC1-TM mRNA expressed, a change in the type of MUC1 isoform produced, an alteration in the epitopes of MUC1 expressed on cell surface and an increase in MUC1 gene product detectable in the circulation.

The Mc5 antibody was generated against delipidated human milk fat globule and recognises the DTRPAP epitope in the VNTR of the MUC1-TM protein (Peterson *et al*, 1995). In the current study the Mc5 antibody recognised membrane associated antigen on mesothelial cells in over half of the effusion samples of nonmalignant origin examined and mesothelioma cells in all of the effusions associated with malignant mesothelioma. Saad *et al* (2005) demonstrated that the choice of antibody clone could greatly influence the accuracy of the use of anti-MUC1/EMA antibodies in distinguishing benign from malignant mesothelial cells in a diagnostic setting. This may be one factor in the debate regarding the role of EMA immunohistochemistry in mesothelioma diagnosis. The E29 clone and four of the other clones examined did not recognise MUC1 expressed on benign mesothelial cells. The E29 clone was also generated against delipidated human milk fat globule and recognises an overlapping epitope (the APDTRP epitope) to that recognised by Mc5. It is noteworthy that the E29 clone stains normal breast, intestine and colon. Alteration in MUC1 glycosylation has been reported in many malignancies (Baldus *et al*, 2004). The current antibody studies suggest that glycosylation of MUC1-TM is altered in malignant mesothelioma cells. However, the actual nature of the alteration is unclear at present. The finding of different staining profiles with the two antibodies, Mc5 and E29, which belong to the same group, recognise the same epitope and are both affected by *in vitro* glycosylation needs to be further investigated.

Levels of MUC1 gene product in serum and effusions can be determined by several tests, the most common being the CA15-3, mucin-like associated antigen, CA27.29 and CA549 assays. Differences between these tests derived from the monoclonal antibodies used to detect MUC1 epitopes and the sensitivity of the antibodies to the level of glycosylation of the protein (Klee and Schreiber, 2004). In the current study, the CA15-3 assay was used. The major clinical role of CA15-3 biomarker is in monitoring breast carcinoma metastases and the evaluation of response to treatment. CA15-3 has previously been found in several small scale studies to be elevated in the serum (Alatas *et al*, 2001) and in effusions of patients with mesothelioma (Miedouge *et al*, 1999; Alatas *et al*, 2001) (Villena *et al*, 2003). In the current study, CA15-3 levels were significantly higher in the serum of mesothelioma patients than healthy controls; however, the finding that levels were elevated in patients with benign lung and pleural disease suggests that serum CA15-3 will not be useful as a diagnostic aid for mesothelioma. Elevated levels of CA15-3 in effusions may be a strong indicator of malignancy in general as previously suggested (Shitrit *et al*, 2005).

The MUC1 gene product detected in the circulation by the CA15-3 assay contains the VNTR region, therefore the protein may be the archetypal MUC1-TM protein that has been released or cleaved from the cell surface, or may be one of the secreted isoforms. There is some evidence to suggest the latter as the MUC1-SEC was detected by PCR in some of the samples derived from mesothelioma patients, but not from normal mesothelial samples or from cells in the nonmalignant pleural effusions examined. Interestingly, the lack of MUC-SEC expression has been associated with ovarian cancer (Obermair *et al*, 2002). Our study used both clinical samples and, in an attempt to limit the influence of nonmesothelial cellular infiltrates, also *in vitro* cultured homogeneous pericardial cells. It was encouraging to find generally a good correlation between the two sample types, as one concern was that alternative splicing of MUC1 might reflect changes induced by culture.

While the secreted splice form was detected in malignant mesothelial cells, it is of particular note that the expression of the full length, VNTR containing MUC1 gene product was 32-fold higher in mesothelioma than in normal cell preparations. Evidence suggests that this overexpression was not due to a gene amplification event, but to an as yet unknown mechanism. MUC1 overexpression is associated with poor prognosis in breast (Rakha *et al*, 2005) and lung adenocarcinoma (Tsutsumida *et al*, 2004); however, its influence in the prognosis of mesothelioma has not been reported.

Various forms of MUC1 are expressed in mesothelioma. We suggest that the specific hypoglycosylated form of the full length tandem repeat containing MUC1 protein product is a useful target for mesothelioma diagnosis, but possibly the expression of novel MUC1 epitopes may represent a potential therapeutic target. Currently there are promising MUC1 directed therapies being investigated in breast, ovarian and non small cell lung cancer. These include therapies targeting MUC1-associated carbohydrates such as sialyl-Tn (STn, Theratope), MUC1-pulsed dendritic cell vaccines and also the MUC1 peptide-based vaccine BLP-25 (Brossart *et al*, 2000; Morse, 2001; Miles and Papazisis, 2003; Wierecky *et al*, 2006a, 2006b). Our findings suggest that the role of MUC1-directed therapy in mesothelioma should be investigated.

## Figures and Tables

**Table 1:** Patient characteristics

**Condition**	**Number of cases**	**Age (Mean±s.d.)**	**Histology or tumour type**
Mesothelioma	60	71.2±11.7	29 epithelial
			16 unspecified
			Nine mixed
			Six sarcomatoid
			
Benign effusions	39	73±13.7	23 exudate
			Seven exudate–infection
			Nine transudate
			
*Controls*			
Normal mesothelial cells	7	64.5±10	
Healthy volunteers	24	48.7±12.8	
Asbestosis patients	11	67.2±11.4	
Asbestosis patients with pleural plaques	21	70.2±12.7	
Other maligancies	26	67.2±12.7	19 lung cancer
			Three lymphoma
			Four other

**Table 2:** Anti-EMA antibodies used in this study

**Clone**	**Source**	**Antigen**	**Epitope**	**Group/Class Staining on T-47D breast cancer cells^a^**	**Cluster^a^**	**Ref**
E29	Dako, Glostrup, Denmark	Delipidated HMFG^b^	APDTRP	Group A–PAN^c^ Membrane & golgi	2	(Cordell *et al*, 1985)
Mc5	Neomarkers Fremont, CA, USA	Delipidated HMFG	DTRPAP	Group A–PAN Membrane & trans-golgi	2	(Peterson *et al*, 1995)
VU2G7	Chemicon Europe	3 × VNTR-galNAc	PDTR	NA	NA	(Ryuko *et al*, 2000)
VU4H5	Santa Cruz, CA, USA	3 × VNTR (nonglycosylated)	PDTR	Group B3–Differentiation dependent^d^ Cytoskeletal-like	2	(Ryuko *et al*, 2000)
CBL263 (VU3C6)	Chemicon Europe	Breast Ca cell line ZR75.1	PDTRPAP	Group B2–Differentiation dependent Golgi >membrane	7A	
MA552	NovaCastra Newcastle upon Tyne	Breast Ca cell line ZR75.1	TRPAPG	Group B1–Differentiation dependent Golgi >membrane	7A	(Baeckstrom *et al*, 1993)
MA695	NovaCastra	Breast Ca cell line ZR75.1	CHO epitope	Group A–PAN Membrane & golgi	7B&C	

a Data modified from workshop reports of the International Society for Oncodevelopmental Biology and Medicine (ISOBM) (Cao *et al*, 1998; Hanisch, 1998).

b HMFG–human milk fat globulin.

c PAN–staining occurred in normal breast, intestine and colon.

d Differentiation-dependent staining in benign breast, intestine and/or colon occurred following periodate treatment.

Cluster 2–reacts with synthetic peptide; the binding is affected by *in vitro* glycosylation; Cluster 7–reacts with carbohydrate or conformational epitope.

**Table 3:** Sensitivity, specificity and false-positive rate of anti-EMA antibodies assessed by immunocytology on confirmed mesothelioma effusions and cases of benign reactive effusions

**Anti-EMA clone**	**Sensitivity**	**Specificity**	**False positives**
VU2G7	5/20 (25%)	16/16 (100%)	0%
VU4H5	5/20 (25%)	16/16 (100%)	0%
CBL263 (VU3C6)	6/19 (32%)	15/15 (100%)	0%
MA552	9/18 (50%)	12/13 (92%)	0%
MA695	14/20 (70%)	5/15 (33%)	0%
E29	16/19 (84%)	14/15 (93%)	0%
Mc5	20/20 (100%)	0/14 (0%)	8/14 (57%)

**Table 4 tbl4:** CA15.3 in serum and pleural effusions

**Sample population**	***n***	**Mean**	**Median**	**Positive (%) (>53 U ml^−1^)**	**Significance level^a^**
*Serum*					
Mesothelioma	49	100±189	47	17 (35%)	
Normal	24	21.5±9.7	21	0	*P*<0.0001
Asbestos-exposed benign disease	32	52±34	38	9 (28%)	ns
Benign effusions	11	34±26	32.6	1 (9%)	*P*=0.016
					
*Effusion*					
PE mesothelioma	51	69±107	24	17 (33%)	
PE benign	30	10±10	8.5	0	ns
PE malignancy	25	66±112	24	7 (28%)	*P*=0.004

a Level of significance relative to samples from patients with mesothelioma.
